# Perceptions of Diseases of Despair by Members of Rural and Urban High-Prevalence Communities: A Qualitative Study

**DOI:** 10.1001/jamanetworkopen.2021.18134

**Published:** 2021-07-23

**Authors:** Daniel R. George, Bethany Snyder, Lauren J. Van Scoy, Emily Brignone, Lawrence Sinoway, Charity Sauder, Andrea Murray, Robert Gladden, Shayann Ramedani, Alana Ernharth, Neha Gupta, Savreen Saran, Jennifer Kraschnewski

**Affiliations:** 1 Department of Humanities, Penn State College of Medicine, Hershey, Pennsylvania; 2 Department of Public Health Sciences, Penn State College of Medicine, Hershey, Pennsylvania; 3 Qualitative and Mixed Methods Core, Penn State College of Medicine, Hershey, Pennsylvania; 4 Department of Medicine, Penn State College of Medicine, Hershey, Pennsylvania; 5 Highmark Health, Pittsburgh, Pennsylvania; 6 Clinical and Translational Science Institute, Penn State College of Medicine, Hershey, Pennsylvania; 7 Penn State College of Medicine, Hershey, Pennsylvania

## Abstract

**Importance:**

Diseases of despair (ie, mortality or morbidity from suicidality, drug abuse, and alcoholism) were first characterized as increasing in rural White working-class populations in midlife with low educational attainment and associated with long-term economic decline. Excess mortality now appears to be associated with working-class citizens across demographic and geographic boundaries, but no known qualitative studies have engaged residents of rural and urban locales with high prevalence of diseases of despair to learn their perspectives.

**Objective:**

To explore perceptions about despair-related illness and potential intervention strategies among diverse community members residing in discrete rural and urban hotspots.

**Design, Setting, and Participants:**

In this qualitative study, high-prevalence hotspots for diseases of despair were identified from health insurance claims data in Central Pennsylvania. Four focus groups were conducted with 60 community members in organizations and coalitions from 3 census block group hotspot clusters in the health system between September 2019 and January 2020. Focus groups explored awareness and beliefs about causation and potential intervention strategies.

**Main Outcomes and Measures:**

A descriptive phenomenological approach was applied to thematic analysis, and a preliminary conceptual model was constructed to describe how various factors may be associated with perpetuating despair and with public health.

**Results:**

In total, 60 adult community members participated in 4 focus groups (44 women, 16 men; 40 White non-Hispanic, 17 Black, and 3 Hispanic/Latino members). Three focus groups with 43 members were held in rural areas with high prevalence of diseases of despair, and 1 focus group with 17 members in a high-prevalence urban area. Four themes emerged with respect to awareness and believed causation of despair-related illness, and participants identified common associated factors, including financial distress, lack of critical infrastructure and social services, deteriorating sense of community, and family fragmentation. Intervention strategies focused around 2 themes: (1) building resilience to despair through better community and organizational coordination and peer support at the local level and (2) encouraging broader state investments in social services and infrastructure to mitigate despair-related illness.

**Conclusions and Relevance:**

In this qualitative study, rural and urban community members identified common factors associated with diseases of despair, highlighting the association between long-term political and economic decline and public health and a need for both community- and state-level solutions to address despair. Health care systems participating in addressing community health needs may improve processes to screen for despair (eg, social history taking) and codesign primary, secondary, and tertiary interventions aimed at addressing factors associated with distress. Such actions have taken on greater urgency with the COVID-19 pandemic.

## Key Points

**Question:**

What do people living in communities with high prevalence of diseases of despair (suicidality, drug abuse, and alcoholism) believe is driving the crisis, and what are their potential solutions?

**Findings:**

In this qualitative study, 60 participants from 3 communities identified factors associated with despair-related disease, including financial distress, lack of infrastructure or social services, deteriorating sense of community, and family fragmentation. Intervention strategies included building resilience through community-level coordination and state investments in social services and infrastructure.

**Meaning:**

Common beliefs were observed in rural and urban communities, highlighting associations between political and economic decline and despair-related illness and suggesting health systems improve clinical processes to screen for despair and address the factors associated with it.

## Teaser

This qualitative study explores the perceptions of people residing in rural and urban areas with high prevalence of diseases of despair regarding potential causes and intervention strategies.

## Introduction

Researchers studying US mortality data from 1999 to 2015 note a precipitous rise in “deaths of despair,” defined as mortality resulting from suicide, drug overdose, and alcohol-related liver disease, especially among working-class midlife adults with low educational attainment.^1,2^ It is theorized^3^ that the long-term labor market decline has weakened family structures, limited access to high-quality health care, reduced participation in social organizations, and caused loneliness and loss of future-oriented hope,^1,2,3,4,5,6,7,8,9^ particularly for those lacking college degrees in post-industrial service- and knowledge-based economies.^10^ In turn, these phenomena may trigger physical, emotional, cognitive, and behavioral changes (eg, chronic pain, anxiety, or depression),^11,12,13,14,15,16^ increasing likelihood of self-harm and substance-use illnesses for working-class Americans.^17^ Risk of converting to despair-related mortality is further increased by distal factors, such as access to handguns, inexpensive alcohol, and prescription or nonprescription drugs (eg, fentanyl, oxycodone).^18,19,20,21^ In 2017, researchers documented 158 000 annual despair-related deaths, with the crisis contributing to downward trends in US life expectancy from 2015 to 2017—the longest sustained decline since 1915 to 1918.^22,23^

Excess mortality disproportionately affects economically distressed regions^24^ that have for decades been reshaped by globalization, deindustrialization, outsourcing,^25,26,27,28^ job automation,^29,30^ deunionization,^31^ and falling real median wages and family incomes.^32,33,34,35^ However, despite initial characterizations of despair-related deaths affecting the White working class in economically distressed rural regions,^1,9,28,36,37,38^ the crisis appears more extensive than previously appreciated.^2,21,32,39,40,41,42,43,44,45^ A 2019 study^46^ found that the increasing death rates among people aged 25 to 64 years from specific causes—drug overdose, suicide, and organ system disease—extended across racial, ethnic, and gender lines, as well as into cities and suburbs (although higher burden remained in the industrial Midwest and Appalachia). Our team previously examined rates of clinically documented diseases of despair (DoD) patterns (ie, comorbidities associated with despair, including suicidal ideation, substance use disorders, and sequela) from 2009 to 2018 and also observed increases across age, gender, and geography (rural or urban).^47^

To better conceptualize this understudied phenomenon, it is necessary to understand what is happening on the ground in communities with increasing despair-related deaths and comorbidities.^17^ However, to our knowledge, no studies have qualitatively examined high-risk locales to assess community perceptions of DoD to generate hypotheses about causation and potential intervention strategies. We used our prior data on clinical prevalence of despair-related illness to identify hotspots in rural and urban settings in our health system’s service area in Central Pennsylvania. Our objective was to explore the influence of DoD in highly affected areas through local focus groups with community leaders to gain insight and consensus into perceptions of the crisis. We felt it important to learn first from community-engaged stakeholders, as opposed to patients, to provide a broader perspective.

## Methods

### Study Population

Claims data from Highmark Inc, a large US-based health insurance company, were used to identify Pennsylvania DoD hotspots. These data included deidentified diagnoses and addresses for members of employer-sponsored, Affordable Care Act, and Medicare insurance plans during 2018. A DoD was defined as a diagnosis related to alcohol or substance use, and suicide ideation or behavior. *International Statistical Classification of Diseases, Tenth Revision*, codes were classified into variables indicating the presence or absence of a DoD (adapted from Healthcare Cost and Utilization Project Clinical Classification Software), including alcohol-related disorders (5.11), substance-related disorders (5.12), and suicide and intentional self-inflicted injury (5.13). This study adhered to the Consolidated Criteria for Reporting Qualitative Research (COREQ) reporting guideline. All procedures were approved by Penn State College of Medicine’s institutional review board. Participants provided oral rather than written informed consent because focus groups were held in conjunction with already existing meetings with time constraints. Participants were offered a chance to ask questions about DoD and received $25 gift cards.

Members were assigned to census block groups based on addresses. For each group, a DoD rate was computed by dividing the number of unique members with a qualifying diagnosis during that year by the total number of members. These rates, along with census block group identifiers and member counts, were then visually represented in an interactive map, which was used to filter areas in the surrounding counties based on overall DoD counts and rates. Each targeted area featured blocks with top deciles of DoD burden.

### Qualitative Approach

Qualitative inquiry is well suited for exploration of complex, multifaceted issues,^48^ such as DoD. Our qualitative approach enabled us to gather consensus information from community members and leaders to generate hypotheses and strategies for addressing the crisis. We used an ontological philosophical assumption appropriate when asking “What is the nature of reality?” (in this case, DoD in hotspots). To examine this question, we used a phenomenological approach and applied a descriptive thematic analysis that is useful when trying to understand individuals’ common, lived experiences regarding a phenomenon.^48,49^ Finally, we explored themes and their associations to construct a preliminary conceptual model describing how various factors perpetuate despair and may influence public health.

### Recruitment for Focus Groups

**Figure 1.**
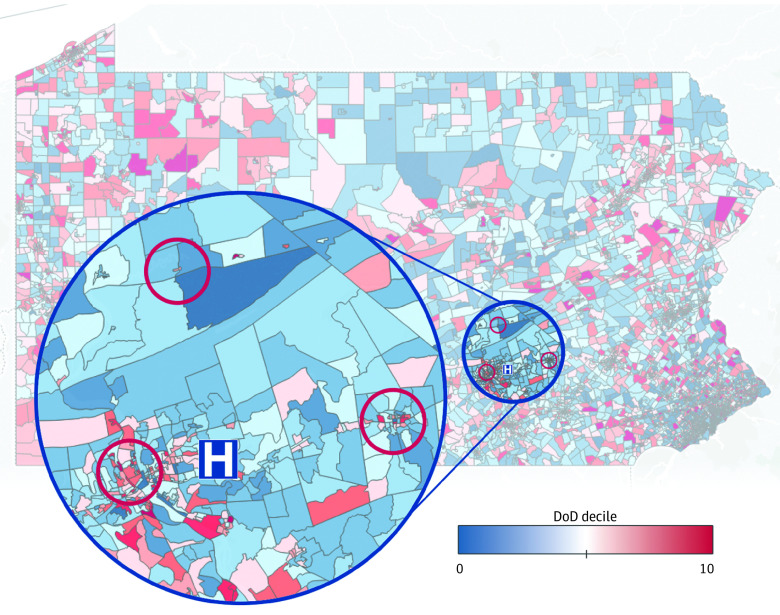
Focus Groups in Diseases of Despair (DoD) Hotspot Locations in Central Pennsylvania Within our hospital system in Central Pennsylvania (H), 2 focus groups were held in high-prevalence census blocks in Dauphin County, Pennsylvania—an urban group in Harrisburg (left red circle) and a rural one in Elizabethville (top red circle). Two rural focus groups were held in the town of Lebanon in Lebanon County, Pennsylvania (right red circle).

Participants in identified DoD hotspots in Dauphin and Lebanon counties (Figure 1) were recruited through established community organizations and coalitions. All participants were community members or community health workers involved in health system outreach who were known to interface with individuals at risk for suicide, alcoholism, or drug use. Persons contacted as part of this purposive sample were (1) older than 18 years of age; (2) English speaking; and (3) formally affiliated with organizations (eg, suicide prevention, homeless shelters) in hotspot communities. Participants were invited to serve in focus groups in conjunction with regularly scheduled meetings with health system outreach personnel. A focus group method was chosen given that it is a useful approach for ascertaining group experiences, achieving consensus about a topic, and facilitating cooperation and interaction among interviewees to yield the most accurate and complete data. Because our research question sought to explore community members’ perceptions of the DoD phenomenon and gain consensus around causation and strategies to address the crisis, focus groups offered a valid, feasible approach.^48^

### Qualitative Data Collection and Rigor

Details of the study are described in eAppendix 1 in the Supplement. In brief, the analytic team consisted of a medical anthropologist (D.R.G.), qualitative research methodologist (L.J.V.S.), and 2 master’s-trained research assistants with experience in qualitative research and community-engaged focus groups (B.S., A.M.). Reflexivity statements addressing researcher positionality are provided in eAppendix 1 in the Supplement. One-hour focus groups, each with 12 to 17 participants, were held from September 2019 to January 2020. Because the DoD crisis has been interpreted along the discrete demographic lines summarized, participants were asked to self-report race/ethnicity and gender. Focus groups were audio recorded and followed a structured interview guide (eAppendix 2 in the Supplement) that was developed after comprehensive literature review. In brief, the guide explored 2 concepts: (1) general awareness and beliefs about causation and (2) intervention strategies. The guides were pilot tested with community members prior to the study.

### Thematic Analysis

Audio recordings were transcribed verbatim. A descriptive approach to thematic analysis was used to analyze transcripts using qualitative software (NVivo 12). A preliminary codebook (eTable in the Supplement) was created by 3 trained qualitative analysts (D.R.G., B.S., and L.J.V.S.) who reviewed transcripts and recorded emerging concepts and codes. Analysts reviewed transcripts until data saturation (when no new codes emerge) was achieved (after 3 transcripts). Four analysts (S.R., A.E., N.G., and S.S.) used the codebook and applied constant comparison method to analyze data.^50^ To assess interrater reliability of coding, the Cohen *κ* coefficient was calculated. When a value of 0.67 for *κ* was achieved among 4 analysts, the remaining data were distributed between and coded by 2 analysts each (pairs). Throughout the process, coding meetings were held to iteratively discuss discrepancies; all codes were adjudicated by a third analyst (L.J.V.S.). Then, coding patterns and frequencies were reviewed by 2 analysts (D.R.G. and B.S.) informing development of themes. Final themes were analyzed at location and hotspot levels to determine similarities, patterns, and differences between discrete locations. A narrative approach was used to describe qualitative themes relative to different group locations and the entire data set. Finally, a conceptual model was developed to illustrate patterns and associations of themes and subthemes.

## Results

### Participant Characteristics

In total, 44 women and 16 men participated in focus groups (40 White/non-Hispanic, 17 Black, and 3 Hispanic/Latino race/ethnicity). Of them, 43 participants lived or worked in rural communities, and 17 participants lived or worked in urban settings (Table).

**Table.** Demographic Characteristics of 60 Patients

Characteristic	Patients, No. (%)				
	Harrisburg, urban	Upper Dauphin, rural	Lebanon (health care workers), rural	Lebanon (community members), rural	Total (%)
No.	17	12	20	11	60 (100)
Gender					
Male	4 (24)	6 (50)	5 (25)	1 (9)	16 (27)
Female	13 (76)	6 (50)	15 (75)	10 (91)	44 (73)
Race/ethnicity					
Black	14 (82)	0	2 (10)	1 (9)	17 (28)
White/non-Hispanic	2 (12)	12 (100)	17 (85)	9 (82)	40 (67)
Hispanic or Latino	1 (6)	0	1 (5)	1 (9)	3 (5)

### Themes

Six themes emerged from 2 categories: awareness and causation of the DoD phenomenon (4 themes), and intervention strategies (2 themes). These themes were similar across focus groups regardless of rural or urban status and are thus presented in aggregate.

### Awareness and Causation of the DoD Phenomenon

#### Theme 1

Participants cited ongoing cycles of financial distress driving instability that worsened mental health and increased risk for drug or alcohol abuse. Both in rural and urban hotspots, participants identified long-term financial strain driving despair in their communities. This strain was commonly attributed to US domestic policies that perpetuated economic disparities for disadvantaged groups while failing to provide secure livelihoods and material protection for working-class residents.

“Rich get richer, poor get poorer. We’re in the middle of it now, and it’s happening very quickly. Folks that’s on the wrong side of that, it gets bigger and bigger—doesn’t matter how hard they work. They will not have the living wage. A full-time job with benefits is very elusive for low-income folks.”

“So many people I’ve talked to with addiction—everything changed from family, to living, to jobs—but it's easier to get a $10 bag of heroin or meth than spend $50 at the grocery store. ‘Cause it’s more expensive to eat than to get high. And the loss of life, they just don’t care anymore. They’d rather be high and happy than not high and sad.”

A subtheme outlined how financial distress dictates patient access to mental health care.

“Over the last 30 years there’s been a great increase in economic disparity. Especially in rural communities where there already weren’t a lot of economic opportunities to begin with … you start chipping away at them, people start not going to the dentist or getting mental health care they need because maybe they can't afford [and] access it.”

“It’s families choosing to see a therapist or buy their groceries.”

Participants pointed out that financial distress, when combined with high medication expense, led many to pursue less expensive chemical solutions to despair, often precipitating longer-term alcohol or substance abuse.

“You can go to the liquor store, buy a $4 bottle of something versus it’s gonna cost me $50 to fill this medication.”

“People who can’t afford their pain medication is going into heroin because it’s cheaper.”

So too did participants identify a criminal legal system producing overincarceration while failing to provide practical support for those with records who face distinct challenges leading to financial distress. Such individuals often confronted difficulties (eg, finding employment, obtaining driver’s licenses) limiting reintegration.

“A lot of our young men and women have records. And they can’t get jobs. People wanna work but soon as they say they got a criminal record, it’s ‘well, we can’t find work.’”

#### Theme 2

Participants described lack of infrastructure and access to fundamental resources (eg, jobs, health care, and quality schools) that could mitigate economic dislocation and drug-abuse.

A common theme—especially in rural focus groups—was that lack of infrastructure led to reduced job stability and compromised health care access. Participants consistently cited lack of public transportation as failing to connect working-class residents with remaining regional manufacturing jobs. Consequently, people continue to settle for part-time or contract jobs with less robust benefit structures (uninsured or underinsured workers) and low wages.

“There’s lots of manufacturing jobs up here, but none of our clients can get to them. I see a lot too with people, there’s just not the kind of benefits with jobs, even full-time jobs, as there used to be, even 10 years ago.”

Participants commonly noted how lack of public transportation also resulted in limited access to health care or mental health care resources.

“That affects health care as well. I’ve needed to set up … services for chronic health concerns, preventative care. If they can’t get there they’re not gonna do preventative health care.”

Beyond transportation, participants identified shortages of hospitals and of mental health and addiction treatment facilities, especially in rural areas, which exacerbated psychological distress.

“Some people do need access to mental health care because I know, people among work and my own family, … if they want to schedule appointments with mental health providers, it’s like, well, this is 2 months in advance. So, really disheartening to wanna reach out and you’re willing to get help, but just can’t get it.”

Participants also described educational systems in economically declining regions insufficiently preparing students for long-term success, increasing risk for long-term despair. This theme was especially prominent in the urban focus group, with participants expressing that the gradual disappearance of school-based vocational training programs meant individuals who didn’t attend college were lacking trades or skills to be self-sufficient in 21st-century job markets and at risk for economic dislocation.

“We don’t have trade school for kids like we used to…. They need a trade, or GED … or they drift.”

Participants also expressed that the inability of overburdened, unprepared teachers to compensate for students’ cumulative disadvantages put children at risk for substance abuse.

“A lot of teachers aren't culturally aware of kids coming from broken homes—how to address dad being incarcerated and mom’s on heroin—so they don’t know how to address kids’ needs to allow them to succeed…. [K]ids are just swapping pills at school. We’ve had kids on our caseload young as 12 on heroin, meth.”

#### Theme 3

Participants described deteriorating sense of community, as well as increasing loneliness and alienation associated with poor mental health and self-harm or suicide.

All focus groups identified declines in civic trust. Participants described decades-long reductions in organizational presence and neighborly interaction and reciprocity, an isolating dynamic that produced growing distrust, loneliness, and lack of shared identity, ultimately affecting mental health.

“If you’d ask my next-door neighbor my son’s name, what he wanted to do, where he wanted to go, they’d have no idea. Because we don’t talk to each other or help each other like we used to. And if we think decades back, we hung out with neighbors every night…. It [isolation] impedes people asking for help.”

“Right now, not just the community, but just the generation, no one trusts anyone…. That promotes isolation.”

Exacerbating this decline was the rise of social media. Participants felt online networks were deleterious both in serving as unfulfilling replacements for embodied human relationships and in worsening mental health.

“Sometimes human connection feels a little lost in this day and age of technology and electronic devices … can’t take the place of human connection. Sometimes that can create a very lonely situation.”

“I was gonna say social media as well because there’s a perception of everyone else having this happy, fabulous life all the time…. I could see that’d make others feel depressed.”

#### Theme 4

Participants reported increasing dysfunction and fragmentation in family life leading to impaired biopsychosocial development, and ultimately to material deprivation.

There was a sense that family breakdown had contributed to growing despair. Participants attributed this to economic stress on 2-earner families, pressures many parents face in caring for children or elder relatives, and the deteriorating sense of community support alluded to in theme 3.

“Increasingly there’s less and less family support. Not even to mention friend support. More and more, folks are pretty much just by themselves, and it seems to be increasing.”

Other explanations focused on absentee parenting or personal failures to discipline children, encourage self-sufficiency, or instill values for productive adulthood.

“The respect level younger kids have for adults now—they don’t have any. And a lot of times kids are raising themselves instead of parents.”

### Intervention Strategies

#### Theme 5

Participants cited diverse solutions to despair at the local and community level. When prompted to think about solutions, participants recommended community-level actions to build resilience to despair. Although many solutions involved actions taken by organizations such as churches, clinics, and nonprofits to address underlying factors associated with distress, other solutions focused on increasing peer support (eg, ride sharing to jobs). Recommendations centered around subthemes, including strengthening communication, relationship building, and fostering greater resiliency in struggling individuals.

“We have to make sure—especially with our youth—we’re doing those core communications we used to do, and don’t lose that because it’s leading to some of these diseases of despair.”

“There’s a parent support-network group starting to grow. If you bring people to the table, you’re offering some incentive/education component, they can start building relationships, sense of community.”

#### Theme 6

Participants desired broader state-level investments in social services and infrastructure. There was also recognition localities could not “go it alone”—that state intervention would be necessary to address underlying despair. Participants felt a key solution involved holding leaders accountable for serving constituencies rather than special interests.

“We have to hold elected officials accountable…. They are the ones who make the law, who determines what’s gonna happen in your community. So we need to either hold them accountable or get rid of them. It’s showing there’s power in numbers.”

There was strong desire to pressure officials to marshal resources to treat systemic failures associated with despair—broadening access to health care and mental health care, transportation infrastructure, job-training centers, and living-wage jobs. Another subtheme concerned a need to strengthen regulatory frameworks that had allowed pharmaceutical companies to market prescription opioids as nonaddictive and flood communities with painkillers that were implicated in many despair-related deaths and illnesses.

“The company that made the drug didn’t get punished [or] put warnings on it for these pain pills…. They wanted [people] to get addicted, make some money off them. And they made like $100 billion and only got fined $1 billion. So that wasn’t enough.”

### Conceptual Model

**Figure 2.**
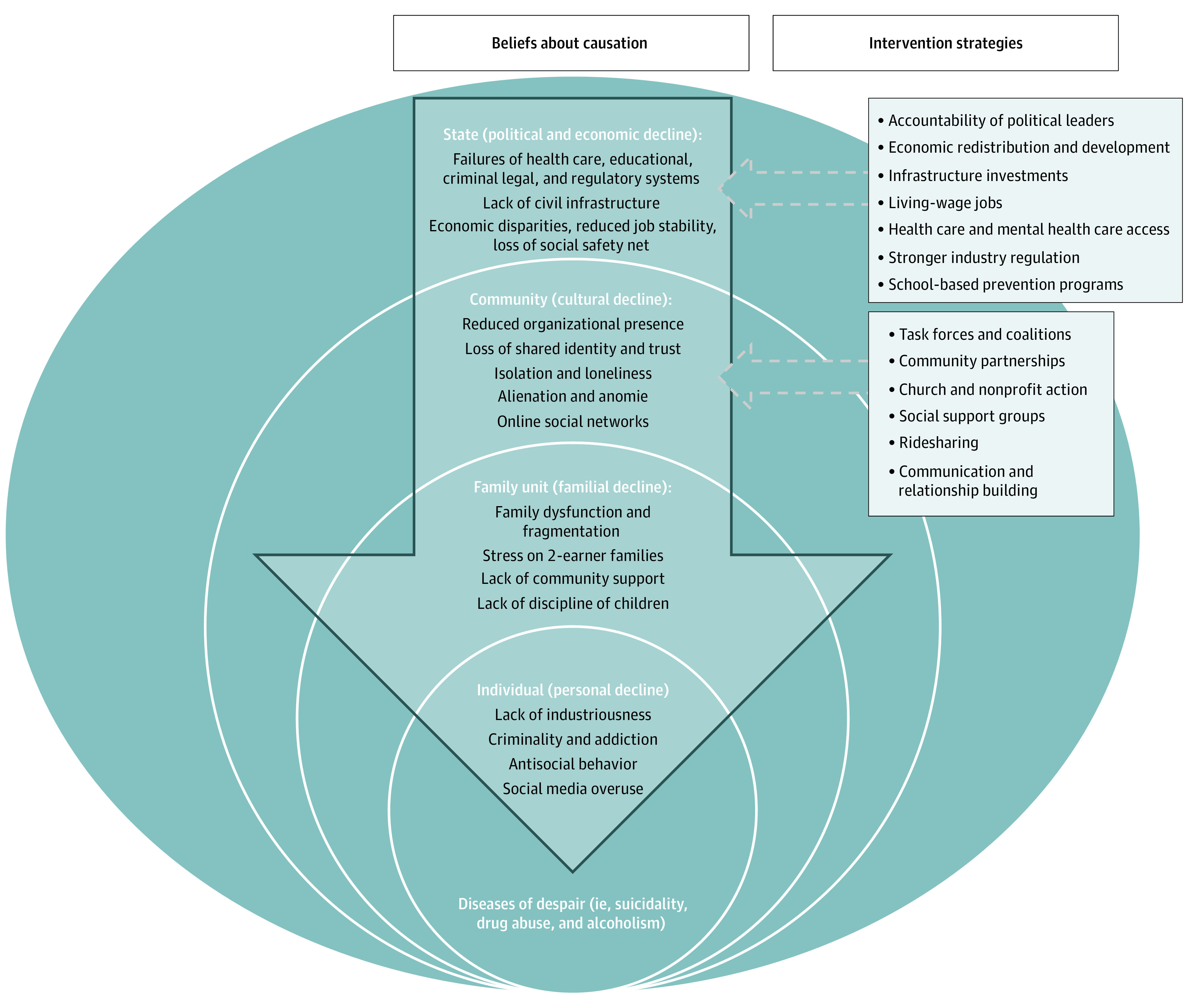
Conceptual Model: Theorized Factors Associated With Diseases of Despair Our conceptual model shows how participants identified state-level failures during the last several decades that initiated subsequent failures at the level of community (cultural decline), family (familial decline), and the individual (personal decline), placing people at greater risk for despair-related illness. Intervention strategies are focused exclusively at the level of state and community.

From these themes, a conceptual model emerged (Figure 2). Participants identified state-level failures during recent decades as the main factor associated with DoD. This political and economic decline was perceived to initiate a cascade of failures at levels of the community (cultural decline), family (familial decline), and individual (personal decline), placing people at greater risk for despair-related illness. Intervention strategies were concentrated at state and community levels, with participants anticipating resilience strengthened through accountable state investments in social services and infrastructure and improved local community and organizational coordination.

## Discussion

This is the first study, to our knowledge, to qualitatively examine perceptions of the DoD phenomenon in high-prevalence rural and urban hotspots. Understanding these perceptions and how the crisis is associated with public health is critical to pursuing state- and community-based interventions. We found consistent beliefs about causation in rural and urban communities, strengthening growing evidence that the DoD crisis is intimately associated with long-term political-economic decline reducing working-class health and material security. Moreover, our findings in a diverse sample further substantiate DoD as broadly associated with Americans beyond the rural White demographic characteristics initially characterized.^1^ It is perhaps notable that participants linked incarceration and DoD because associations between mortality rates and jail incarceration have been recently documented^51,52^ and may deserve further inquiry. Participants frequently discussed how youth were affected by rising despair, suggesting value in examining adverse childhood experiences^53^ data as predictive of DoD risk, and using data to guide delivery of early-childhood and school-based intervention programs shown to decrease despair-related behaviors.^54^

Our findings suggest that state-level interventions identified by participants (eg, economic development and redistribution; infrastructural investment; social services aimed at alleviating factors associated with despair and precarity, such as health care, education or vocational training, access to food and housing; living-wage jobs with time for meaningful family and community life); and tighter pharmaceutical industry regulation would cumulatively lower DoD risk, especially in conjunction with community-level initiatives (eg, task forces and nonprofit services). Indeed, community leaders and organizations play critical roles in addressing health disparities through established relationships with members and through the ability to support trusting relationships with health care systems.^55^ Our findings also have implications for clinical practice and health care administrators, including opportunities for improved social history taking to screen for despair-related illness.^56,57^ Although time-consuming, better processes for systemically identifying and tracking risk factors for despair may allow targeted clinical interventions to mitigate progression to mortality.^17^ Given that being uninsured or underinsured appears to place vulnerable populations at greater risk for financial and mental distress, health care systems may need to go beyond improving clinical processes and codesign primary, secondary, and tertiary interventions to address underlying factors associated with despair and precarity.^47^ Such interventions may require community partners not typically included in conventional health services (eg, community health workers and social workers). Building and evaluating effective organizational partnerships and best practices in integrated care of at-risk patients would be valuable.^47^

The DoD crisis has taken on greater urgency with the COVID-19 pandemic, with socioeconomic stressors exacerbated by the pandemic projected to cause more than 150 000 additional cases.^57,58^ It will be important to examine how despair is affected by prolonged isolation, loss of jobs or benefits, breakdown of social protections, minimal treatment of comorbid chronic and mental health conditions, and drug or domestic abuse.^59^ Research can establish how communities view health systems and clinicians as partners in the crisis, and what best practices are ameliorative (eg, clinical screening for risk and resource navigation, improved telemedicine). Given increasing evidence for structural factors such as economic deterioration associated with DoD,^3^ big-data approaches may be useful in comprehensively examining socioeconomic patterns in hotspots over decades and their associations with health.

### Limitations and Strengths

This study had several limitations. The analysis engaged participants affiliated with a single hospital system’s outreach, which may have led to selection bias. Relatedly, DoD hotspots were identified through insurance claims, limited to persons accessing care and providing accurate residences; this approach may have undercounted DoD, particularly in areas with high levels of underinsured. Although participants were familiar with people affected by DoD, they were often not personally afflicted. Moreover, although groups were demographically diverse overall, there was less intragroup diversity, and two-thirds of participants were women. Given that men have been disproportionately affected by DoD,^1^ future studies may aim for greater gender balance. Limiting focus groups to English speakers may have excluded representation from other vulnerable cohorts, and only 1 of 4 groups was held in an urban setting. Moreover, disproportionate focus on community-oriented solutions may have been associated with participants’ roles in community groups that interface with the health care system.

Despite these limitations, the study had numerous strengths. Our population was a large, multisite cohort of 60 diverse individuals purposively sampled to capture multiple perspectives in rural and urban hotspots. Interviews and analyses were performed with methodologic rigor, and this study represents the first attempt, to our knowledge, to qualitatively elucidate lived experiences in communities with high risk for DoD.

## Conclusions

Common beliefs about DoD were observed in rural and urban communities, highlighting associations between political and economic decline and despair-related illness and identifying diverse intervention strategies. Health care systems may improve timely DoD detection through targeted screening and address underlying factors associated with distress, particularly during the COVID-19 pandemic era.

## Notes

Supplement. **eAppendix 1.** Description of Adherence to the 32 Items of the Consolidated Criterion for Reporting Qualitative Research (COREQ) **eAppendix 2.** Focus Group Interview Guide **eTable.** Qualitative Codebook and Code Definitions
