# Supplementary material for: Perceptions of Diseases of Despair by Members of Rural and Urban High-Prevalence Communities: A Qualitative Study
Source: JAMA Netw Open. 2021 Jul 23;4(7):e2118134. doi: 10.1001/jamanetworkopen.2021.18134 (PMC8303097; doi:10.1001/jamanetworkopen.2021.18134)
Supplement: Supplement. — eAppendix 1. Description of Adherence to the 32 Items of the Consolidated Criterion for Reporting Qualitative Research (COREQ) eAppendix 2. Focus Group Interview Guide eTable. Qualitative Codebook and Code Definitions [file jamanetwopen-e2118134-s001.pdf]

Supplementary Online Content

George DR, Snyder B, Van Scoy LJ, et al. Perceptions of diseases of despair by members of rural and urban high-prevalence communities: a qualitative study. *JAMA Netw Open*. 2021;4(7):e2118134. doi:10.1001/jamanetworkopen.2021.18134

eAppendix 1. Description of Adherence to the 32 Items of the Consolidated Criterion for Reporting Qualitative Research (COREQ)
eAppendix 2. Focus Group Interview Guide
eTable. Qualitative Codebook and Code Definitions

This supplementary material has been provided by the authors to give readers additional information about their work.

© 2021 George DR et al. *JAMA Network Open*.

eAppendix 1. Description of Adherence to the 32 Items of the Consolidated Criterion for Reporting Qualitative Research (COREQ)

Domain 1: Research Team and Reflexivity Statement

Relationship with focus group participants was established by the Penn State Clinical and Translational Science Institute Community Engaged Research Core through its regular meetings with community members and health workers loosely affiliated with the health system. Participants had met the interviewer (AM) prior to the focus group, as she had previously led community meetings to discuss extant health issues.

The qualitative research team reflexivity statements are as follow.

> The lead qualitative researcher (DRG) has a PhD in Medical Anthropology and is an Associate Professor of Humanities and Public Health Sciences. He has an interest in Diseases of Despair (DoD) and social determinants of health, and a belief in the material (rather than cultural) basis of the DoD crisis, and this predilection for examining the structural factors underlying human phenomenon influenced the design and analysis of the study and is reflected in the interpretation of final themes. Moreover, the researcher has a medical humanities background and applies a humanistic perspective to his evaluation of data. The researcher took seriously the need for objectivity during design and analysis as a result of potential implicit bias in other team members with connections to the topic. He bracketed bias to the extent possible by having a community member from the first focus group and colleagues external to the study (BS, LVJ) vet the interview guide and lead the qualitative analysis, and by enlisting another member of the research team (AM) to facilitate all focus groups.
>
> A second analyst (LJV) is a physician scientist whose research interest focuses on end of life and communication issues. Her experiences with medicine and research give perspective into the meaning behind disease burden of the Diseases of Despair. She is the co-director and co-founder of the Qualitative and Mixed Methods Research Core at the home university. Her qualitative work generally follows a pragmatic approach using descriptive methods to understand phenomena, and she applies a humanistic perspective to data analysis. She has not conducted research on social determinants of health in the past. She had no stake in the success of the project and was brought in as a methodologic consultant. Philosophically, she believes people make meaning through their experiences and chose behaviors based on their emotions and rational problem-solving skills. Therefore, she values lived experience and emotional connections while analyzing qualitative data, and this perspective is reflected in the analysis of the themes in this paper. She took seriously the need for objectivity during design and analysis as a result of potential implicit bias in other team members with connections to the topic. This researcher bracketed bias to the extent possible by consistently checking methodologic and analytic decisions by referencing the raw dataset.
>
> The focus group facilitator (AM) for this study was a female Community Engagement Coordinator for the Penn State Health system with a Master's in Public Health with 5 years of experience in community-engaged research and qualitative inquiry. She was formally trained in qualitative research methodology by West Chester University of Pennsylvania. She developed a relationship with focus group participants through prior work as part of the Penn State Clinical and Translational Science Institute's Engaged Research Core, which develops collaborative partnerships within the health system's service area. The interviewer has a personal history of interest in rural health and racial and ethnic minority health and social determinants of health. While this researcher herself has not been affected by diseases of despair her hometown has been affected by these issues, imparting an emotional connection to the topic and potentially influencing her facilitation of the focus groups. Objectivity was pursued to the extent possible by using an IRB-approved interview guide developed by the research team, with input from community members.
>
> The research assistant who assisted with coding (BS) was a research project manager and research technologist for a research core specializing in qualitative and mixed methodology at the time the data was

© 2021 George DR et al. *JAMA Network Open*.

analyzed and manuscript was written. The author acknowledges her bias and preference for this methodology, and thus the methodology presented in this paper. The author had little to no background knowledge on the academic work related to Diseases of Despair or its associated theoretical framework at the time of developing the paper. The author's expertise was related to facilitating and guiding the qualitative coding team throughout the coding of the focus group data, and applying a systematic approach to interpreting the qualitative data and reporting the results. The author's role was to facilitate a methodologic approach along with the team's content expert (DRG) to develop themes and interpretations that were sound, rigorous, and had fidelity to the focus group data. Objectivity regarding the data was prioritized, and the author took special care to analyze the data iteratively, grounding the themes to the data throughout multiple stages of the analysis process.

The other coders were medical students and only coded using NVivo but did not analyze the data.

Participants were invited to serve in focus groups (after providing informed consent) in conjunction with regularly scheduled meetings with health system outreach personnel. A relationship was established through AM prior to study commencement. Participants were informed of the goals of the research via IRB approved scripts prior to focus group sessions. Since the interviewer was known, nothing was specifically reported with regard to bias, assumptions, and interests in the research topic.

Domain 2: Study Design

We used an ontological philosophical assumption appropriate when asking 'What is the nature of reality?' (in this case, DoD in hotspots). To do so, we used a phenomenological approach and employed a descriptive thematic analysis that is useful when trying to understand individuals' common, lived experiences regarding a phenomenon.[48,49] Finally, we examined themes and their relationships to construct a preliminary conceptual model describing how various factors perpetuate despair and affect public health. Additional details of our analytic approach are provided in the manuscript body.

Participants were selected based on their residence/community-based work in three high-prevalence hotspots that were identified using insurance claims data for diseases of despair. This process is described in detail in the main body of the manuscript. We used purposive sampling based on these hotspots to select communities with census blocks featuring high deciles of DoD burden, and conducted a focus group within each area through existing community partnerships. Present at the focus groups were the research facilitator, and three medical students who took initial notes on the sessions. Four, one-hour focus groups were held in urban (n=1) and rural (n=3) hotspots. Those who chose not to participate in the focus group were not present. The sample is described in the main manuscript.

The interview guide is provided in Supplement 1 (below). Repeat interviews were not performed. All focus groups were audio recorded. The facilitator and medical students took field notes to help guide the one-hour discussions, but these notes were not intended for incorporation into the analysis. Data saturation was judged to occur after review of three transcripts. Transcripts were not returned to participants due to feasibility issues.

Domain 3: analysis and findings

There were four data coders and two senior analysts who reviewed the data. The coding tree is provided below (in the eTable). The constant comparison method was used during the analytic process to derive themes that emerged from the data and were not identified in advance. Coding was performed using NVivo 12. Results were reviewed by a member of the Harrisburg (urban) focus group as a means of ensuring that the results were truly reflective of the participants' experiences. Major themes and quotations are presented in the manuscript. Minor themes and outlier cases (e.g., participants who argued that diseases of despair are a result of failures in personal responsibility) are also reported.

© 2021 George DR et al. *JAMA Network Open*.

eAppendix 2. Focus Group Interview Guide

General awareness and beliefs about causation

- Please raise your hand if you are familiar with the concept of "diseases of despair".
    - [If yes], how did you become aware of it, and what does it mean to you?
    - [If no], when you hear the term "diseases of despair", what does it mean to you?

- What do you think may be driving deaths and illness from suicide, alcoholism, and drug abuse?
    - Have you noticed these problems in your community? Tell me more about that.
    - What conditions have changed in your community over the past several decades? How might this have made an impact where you live?
    - What are your thoughts about the emotional well-being of your community?
    - How do you see mental health playing a role in the problems we're seeing in our communities today?
    - What do you feel makes some people/organizations within your area more resilient to despair? How do you think we can we build on this?

- As we are talking about "Diseases of Despair", are there any individual stories that come to mind? People who you are reminded of—either with respect to being the victim or being particularly resilient to them? (*Moderator: please remind participants to de-identify any stories*)

Intervention strategies

(Moderator: transition the group from thinking about the problem to thinking about solutions)

- With respect to solutions, how is your organization/community addressing despair-related issues in your patients/clients?
    - What have you done well?
    - What haven't you done well?
    - Are there specific resources that have helped you in addressing these issues?
    - What could you be doing better to build community resilience?
    - Are there strategic partnerships within the community that could help?
    - What major barriers/limitations do you feel your organization faces?

- Beyond your local community, are there other interventions that might help address resilience to diseases of despair?

© 2021 George DR et al. *JAMA Network Open*.

eTable. Qualitative Codebook and Code Definitions

Category	Code	Definition
Addiction	Access to addictive substances	Participant reports access to opioids (heroin, oxycodone, hydrocodone, morphine, etc.) and/or access or availability of inexpensive alcohol.
	Corrupt practices of drug companies leading to excess availability of opiates	Participant reports misconduct, corrupt practices of drug companies themselves, e.g., overproduction of addictive pharmaceuticals, mis-reporting of addictiveness of pharmaceuticals, etc.
	Using illicit drugs as a replacement for pharmaceuticals	Participant reports the practice of individuals using illicit drugs as a replacement for addictive pharmaceuticals because they may have better access to illicit drugs.
	Other	Participant discusses information about addiction that is not otherwise fit into another code.
Community decline; decreased cohesiveness; loss of virtue; moral decay leading to diseases of despair (at the individual, societal, or institutional level)	Apathetic approach to life at the personal level	Participant reports individuals in the community do not want to work and accept living off of unemployment, lack of motivation for working, etc. Diminished work ethic; motivation for holding a job. Participants reports either themselves or other individuals lack a vision or goal of success for themselves.
	Change in familial roles, dynamics, responsibilities, structure over time	Participant reports changes over time in child discipline, single parenthood, non-involved parents, compounding familial roles and expectations, stressed family dynamics, etc.
	Community identity; sense of increasing 'outsiders'	Participant reports a sense of ‘us vs. them’ mentality, references to “community culture”, changes in generational norms, loss of a sense of security in the community.
	Other	Participant notes a loss of trust or fellow-feeling in the context of neighborhoods.
Epidemic of loneliness (individual level)	Alienation, loneliness	Participant reports loneliness/isolation, non-meaningful relationships, physical isolation, etc at the individual level, either in reference to themselves or another individual.
	Role of social media and technology; Mental health; exacerbating alienation and loneliness	Participant reports role of technology in deepening loneliness/isolation/alienation.
	Other	Participant references an earlier time when people were less lonely and more civically engaged.
Failure of educational system	Lack of preparation or opportunities for higher education or lack of skilled labor training or schools	Participant reports lack of vocational schools, lack of education for practical jobs. Participant reports limited opportunities to pursue college degrees, young people being deterred by cost of education, etc.
	Lack of cultural sensitivity in schools	Participant reports teachers from outside the communities they teach in. Teachers’ lack of understanding of their students’ lived experiences.

© 2021 George DR et al. *JAMA Network Open*.

	Other	Participant discusses a sense that the way students are raised increases their risk for truancy/misbehavior.
Failure of government	Criminal legal system	Participant reports over incarceration, a broken system, a system that sets people up to fail, minorities being disproportionately incarcerated, criminal records as impediment to employment, etc.
	Lack of accountability, lack of regulatory or policy oversight	Participant reports the government is not accountability for its decisions. Participant reports lack of policies and/or regulations in place to protect citizens.
	Lack of civil infrastructure (e.g., housing, transportation, day cares)	Participant reports lack of public transportation, hospitals, mental health facilities, day cares, etc. Participant reports lack of affordable adequate housing (housing crisis).
	System cuts, unequal access to social services	Participant reports overburdened social service system, sub-optimal social services, high caseloads, etc. Participant reports unequal access to social services (e.g., rural vs better access in urban areas).
	Other	Participant reports imbalances/disparities in distribution of societal resources.
Finances	Debt	Participant reports housing, student loan debt or any other kind of debt.
	Poverty	Participant reports they or others can't afford basic needs (e.g., co-pays, medications), struggles of low wages, etc.
	Other	Participant links discreet behaviors (e.g., drinking) to financial distress.
Global economic shift	Economic stagnation for working class people; traumatic economic events	Participant reports the housing crisis, the great recession, etc.
	Increase in economic disparity	Participant reports the wealthy getting wealthier, growing gap in wealth, etc.
	Lower quality of job-related benefits, rising demands in work productivity	Participant reports full-time workers not having job related benefits such as health insurance, retirement investments, paid time off, etc. Participant reports increasing employer demands for productivity in the workplace.
	Other	Participant reports life being on-the-whole more difficult for workers than it was in past decades.
Healthcare system	Capacity for treating mental illness	Participant reports lack of resources for mental illness, access to help, etc.
	Expense, financing, cost of healthcare	Participant reports problems related to un-insured and under-insured individuals, cost of healthcare, etc.
	Other	Participant reports hospital closures or transportation limitations to healthcare facilities.
	Perceptions that self-harm is on the rise, more prevalent as a result of lack of healthcare access.	Participant reports perception of rise in suicides, attempted suicides, liver disease, addiction, overdoses, etc.
Solutions	Community resources	Participant reports churches, cultural diversity events, community health councils, sense of community support as solutions to combat diseases of despair.

© 2021 George DR et al. *JAMA Network Open*.

	State-level actions	Participant reports state or national policies or actions necessary to address underlying drivers of despair.
	Other	Participant reports other individual, group, or governmental action to address root causes of diseases of despair.

© 2021 George DR et al. *JAMA Network Open*.
